# Training Clinic Providers on Advance Care Planning Improves Provider Self-Efficacy

**DOI:** 10.1093/geroni/igab046.2825

**Published:** 2021-12-17

**Authors:** Jeffrey Graupner, Sandy Tun, Carolyn Read, Amena Qureshi, Cassie Lee, Katherine Thompson

**Affiliations:** 1 University of Chicago, Chicago, Illinois, United States; 2 University of Chicago Medicine, Chicago, Illinois, United States; 3 Oak Street Health, Oak Street Health, Illinois, United States; 4 Oak Street Health, Chicago, Illinois, United States

## Abstract

Advance care planning (ACP) is a vital step to ensure patients receive and prioritize the care that best aligns with their end-of-life wishes, including discussion and documentation of an advance directive. Significant gaps in ACP among underserved populations have been well documented. Research suggests a successful strategy for increasing the communication between provider and patient about ACP is to educate clinicians on this important issue. Three, 2.5 hour training sessions were provided to healthcare staff of a large chain of older adult primary care clinics across three states. Lecture materials were created and presented by a palliative care (PC) physician and PC nurse practitioner. Presentations were held both in person and virtually. Participants were asked to complete a pre/post-training survey online which included a validated 17-item ACP Self-Efficacy Scale (Baughman, 2017), perceived barriers checklist, and additional quality improvement measures. A total of 131 providers attended one of three training sessions. 76 providers (58.0%) and 47 providers (35.9%) completed pre- and post-training surveys respectively. Scores on a 17-item validated ACP Self-Efficacy Scale were significantly higher after training (Wilcoxon signed rank test, Z= 4.42, p <.001). Participants ranked “lack of time” as the number one barrier to having ACP conversations both before and after the training, whereas “lack of training” ranked 2nd and fell to 7th after the training. These initial results suggest ACP self-efficacy among providers can be increased through a one-time training session. Previous literature has highlighted the importance of provider self-efficacy as factor in increasing ACP conversations with patients.

